# A Multithread Nested Neural Network Architecture to Model Surface Plasmon Polaritons Propagation

**DOI:** 10.3390/mi7070110

**Published:** 2016-06-30

**Authors:** Giacomo Capizzi, Grazia Lo Sciuto, Christian Napoli, Emiliano Tramontana

**Affiliations:** 1 Department of Electrical, Electronics and Informatics Engineering, University of Catania, 95125 Catania, Italy; gcapizzi@diees.unict.it; 2 Department of Engineering, University of Roma Tre, 00146 Rome, Italy; glosciuto@dii.unict.it; 3 Department of Mathematics and Informatics, University of Catania, 95125 Catania, Italy; tramontana@dmi.unict.it

**Keywords:** nanotechnologies, photonics, nanoplasmonics, neural networks, high performance computing

## Abstract

Surface Plasmon Polaritons are collective oscillations of electrons occurring at the interface between a metal and a dielectric. The propagation phenomena in plasmonic nanostructures is not fully understood and the interdependence between propagation and metal thickness requires further investigation. We propose an ad-hoc neural network topology assisting the study of the said propagation when several parameters, such as wavelengths, propagation length and metal thickness are considered. This approach is novel and can be considered a first attempt at fully automating such a numerical computation. For the proposed neural network topology, an advanced training procedure has been devised in order to shun the possibility of accumulating errors. The provided results can be useful, e.g., to improve the efficiency of photocells, for photon harvesting, and for improving the accuracy of models for solid state devices.

## 1. Introduction

In recent years, the development of nanoplasmonic has been a topic of increasing interest with significant applications, such as, e.g., surface enhanced Raman spectroscopy, photovoltaic devices optimisation, optical filters, photonic band gap structures, biological and chemical sensing [1,2,3,4,5]. This recent progress has been obtained thanks to the advances in nanofabrication technology. Plasmonic nanoparticles are of great interest for light trapping in thin-film silicon solar cells, hence for the manipulation of light confined to small length scales. Therefore, the areas of propagating electromagnetic modes on metallic dielectric interfaces, resonances between the electron gas and surrounding dielectrics, and the interaction of light with metals, are widely studied [6]. In such a field, optical systems made of a metal-dielectric interface are liable to electromagnetic modes called Surface Plasmon Polaritons (SPPs) and make it possible to control light having wavelengths below 100 nm [7,8,9]. SPPs are collective oscillations of electrons occurring at the interface between a metal and a dielectric when a photon couples to the free electron gas of the metal. Some papers have appeared in the literature to describe the simulations and analyses of the excitation and propagation of SPPs on sinusoidal metallic gratings in conical mounting and the bending in metallic nanowires [7,10]. Several papers in the literature have proposed correlation models for SPPs propagation length and excitation wavelength, e.g., [11] describes a correlation model in terms of center-to-center pitch among waveguide relative maxima. However, such proposed models are typically used in triple interfaces (dielectric-metal-dielectric or metal-dielectric-metal. Hence, more studies and investigations are needed due to the actual poor understanding of the propagation phenomena in the plasmonic nanostructures, especially concerning asymmetrical systems such as dielectric-metal structures [12,13].

In this paper, we examine the propagation of SPPs due to the combination of the molybdenum nanostructure in the presence of a dielectric exposed to an electric field and photon fluxes with energies spanning a wavelength range 400 to 700 nm. Therefore, we propose a novel neural network (NN) topology, dubbed Nested Neural Network Architecture (NNNA), assisting us to study the inner relation between SPPs exciting wavelength, metal thickness and SPP wavelength and propagation length at a metal flat interface separating dielectric medium. As a result, by employing such a suitable NNNA topology we can determine the interdependence between SPP propagation and metal thickness. Due to the high sensitivity level of the neural model to data oscillations a novel NN training procedure has been devised in order to avoid polarisations and miscorrections of some NN weights. Moreover, since such a training procedure could be computationally expensive and take a long time, a parallel version using an OpenMP environment, with shared memory, has been developed and optimised to obtain maximum advantage from the available parallel hardware. A big amount of data has been put into proper use for the investigated NN topology. Such data have been made available by solving 3D Maxwell equations with the relative boundary conditions. For the resolution of the electromagnetic field and for the investigations of SPPs characteristics in a simple plasmonic structure, we have been developed numerical calculations and simulations to solve Maxwell’s equation with the Finite Element Method (FEM). This has been performed by using the commercial software packages COMSOL Multiphysics (v 4.3b, COMSOL Inc., Burlington, MA, USA).

**Figure 1:**
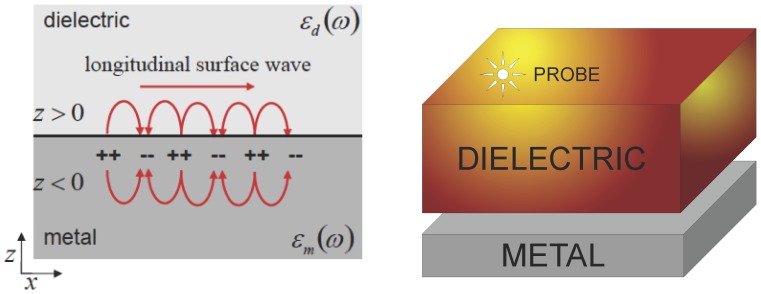
Implemented geometry in COMSOL (**left**), and geometry that models Surface Plasmon Polaritons (SPP) propagation from a metal-dielectric interface (**right**).

**Figure 2:**
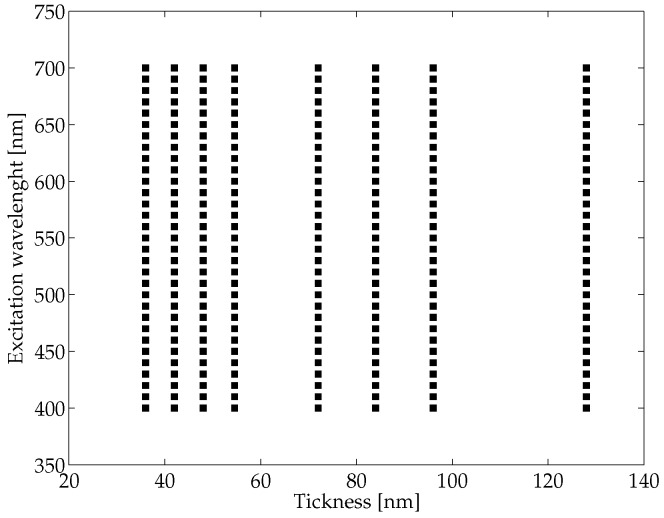
The simulation survey coverage in the features space concerning the excitation wavelength $\lambda_{0}$ and the adopted tickness *t*.

In our simulation, we used a 3D geometry (see Figure 1) to represent the two media (metal and dielectric) and solved the full wave 3D Maxwell equations (using the above said software packages). Then, we have computed the propagation length $L_{SPP}$ and plasmon wavelength $\lambda_{SPP}$ for different thickness conditions. The external surface of the plasmonic structure has been modeled by means of perfectly matched layer boundary conditions and taking into account the monochromaticity conditions for the exciting photon flux. The energy of such photons has been selected within an excitation wavelength, named $\lambda_{0}$, in the range of 400 to 700 nm, therefore compatible with the visible light spectrum. As reported in Figure 2, in our simulation we considered several excitation wavelengths ($\lambda_{0}$) and thicknesses (*t*). For each pair of values $\lambda_{0}$ and *t*, we have computed the corresponding surface polaritons’ wave propagating from the dielectric-metal interface and decaying into the metal.

In the following we will first give, in Section 2, an introduction to SPPs. Section 3 describes how the initial data have been computed. Section 4 explains the proposed novel neural network architecture that models the correlation between each scenario (in terms of excitation wavelength and thickness) to the related SPPs wavelength $\lambda_{SPP}$ and propagation length $L_{SPP}$. Section 5 describes our parallel procedure for training. Finally, Section 6 draws our conclusions.

## 2. Brief Introduction to Surface Plasmon Polaritons (SPPs)

SPPs can only be generated along metal-dielectric interfaces over a wide range of frequencies and evanescently decay in the perpendicular direction of the metal-dielectric interface. Recently, the light absorption by solar cells patterned with metallic nanoparticles has been investigated; however, we consider light-excited SPPs at the metal surface proposing an investigation that can be used to efficiently capture light in solar energy conversion cells [2].

At ultraviolet and visible frequencies, the noble metals (Au, Ag, etc.) acquire a behaviour similar to that of a dielectric, and allow the propagation of electromagnetic (EM) waves with a significant penetration within the metal, where the losses of energy are strongly related to the electronic structure. The scattering properties can be represented by a complex dielectric constant. The losses in the conductor are due to collisions between the free electrons and interband transitions. The optical properties can be explained by the plasma model, where a number of free electrons move against the fixed ions (Drude model). The electrons oscillate in response to the applied electromagnetic field with a characteristic collision frequency and a certain relaxation time, determining the frequency dependence of the dielectric constant [14].

**Figure 3:**
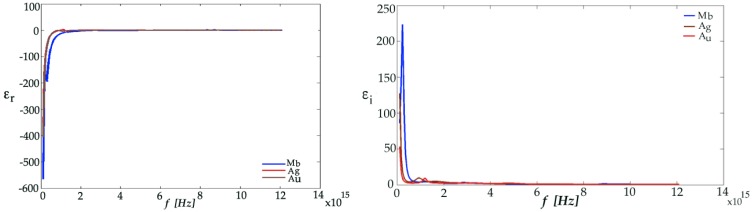
The real part (**left**) and the imaginary part (**right**) of the dielectric constant of various noble metals, such as Au, Ag and Mb.

It was observed that the dielectric constant evaluated as a relative complex for the noble metals with the high negative real part value of the dielectric constant, would assure the existence of the plasmons. The negative real part of the dielectric constant of various metals such as Au, Ag and Mb decreases with the increase of wavelength, while the imaginary part is increased as shown in Figure 3.

The simplest geometry that can generate SPPs is that of a single and flat interface (see Figure 1 (left)) between a dielectric with a real dielectric constant $\varepsilon_{d}>0$ and an adjacent metal described by means of a dielectric function $\varepsilon_{m}\left(\omega \right)$, where $Re[\varepsilon_{m}\left(\omega \right)]<0$.

The following reports the mathematical equations that describe the propagation of SPPs in both the dielectric (*d*) and the metal (*m*). Of course, such equations have same mathematical form, however they differ in the constants describing the medium:

(1) $$\begin{array}{ccc} H_{d} & = & \left(0,H_{yd},0\right)e^{i(k_{xd}\;x+k_{zd}\;z-\omega t)}\\ E_{d} & = & \left(E_{xd},0,E_{zd}\right)e^{i(k_{xd}\;x+k_{zd}\;z-\omega t)}\\ H_{m} & = & \left(0,H_{ym},0\right)e^{i(k_{xm}\;x-k_{zm}\;z-\omega t)}\\ E_{m} & = & \left(E_{xm},0,E_{zm}\right)e^{i(k_{xm}\;x-k_{zm}\;z-\omega t)} \end{array}$$

with boundary condition at z=0, we obtain the following equivalencies:

(2) $$\begin{array}{ccc} E_{xm} & = & E_{xd}\\ H_{ym} & = & H_{yd}\\ \varepsilon_{m}\;E_{zm} & = & \varepsilon_{d}\;E_{zd} \end{array}$$

and finally, as implied, we have the Snell equation:

(3) $$k_{xm}=k_{xd}$$

In this discussion, we have assumed that the interface is normal to the *z*-axis and SPPs propagate along the *x* direction (i.e., $k_{y}=0$), the SPP wave vector $k_{x}$ (or *β*) is related to the optical frequency *ω* through the dispersion relation:

(4) $$k_{x}=k_{0}\;\sqrt{\frac{\varepsilon_{d}\;\varepsilon_{m}}{\varepsilon_{d}+\varepsilon_{m}}}$$

We take *ω* to be real and allow $k_{x}$ to be complex, since our main interest is in stationary monochromatic SPP fields in a finite area, where:

(5) $$k_{0}=\frac{\omega }{c}$$

is the wave vector in free space, and $\lambda_{0}=\frac{c}{\omega }$ is the wavelength in vacuum. For metals, the permittivity is complex, which leads to $k_{x}$ being complex. The imaginary part of $k_{x}$ defines the SPP’s damping as it propagates along the surface. The real part of $k_{x}$ is related to the plasmon’s wavelength, $\lambda_{SPP}$, as:

(6) $$\lambda_{SPP}=\frac{2\pi }{Re[k_{x}]}$$

The SPPs propagation length, $L_{SPP}$, is defined as:

(7) $$L_{SPP}=\frac{1}{Im[k_{x}]}$$

In conclusion of this brief review of SPPs, we report the expression of the electric field of plasmon wave:

(8) $$E_{SPP}=E_{0}^{\pm }\;e^{i(k_{x}x\pm k_{z}z-\omega t)}$$

where $k_{x}=k_{x}^{\prime }+ik_{x}^{\prime \prime }$, $k_{x}^{\prime }=\frac{2\pi }{\lambda_{SPP}}$.

## 3. Input Data for Our Nested Neural Network Architecture (NNNA)

In this paper we investigate a metal-dielectric interface composed of Molybdenum. This structure reduces the computational effort for the investigations on the relation between dispersion and thickness of metal. Even though, this relation is unaffected by the complexity of the structure.

Initially, in order to generate SPPs, we used an electromagnetic plane wave TM-polarised with angle of incidence equal to 45 degrees. The metal-dielectric interface was excited by a probe-antenna, with a size of 1320 nm by 6.6 nm, inserted through a glass film with a refractive index of 1.5 to induce the electric field in the metal/air interface. By solving the full wave 3D Maxwell equations in the simple geometry shown in Figure 1 (left), which separates metal and dielectric, using the finite element method-based software package COMSOL Multiphysics, we have obtained the $L_{SPP}$ and $\lambda_{SPP}$ data values for several thickness values. The perfectly matched layer boundary condition was chosen for the external surface of the plasmon structure.

The values of $L_{SPP}$ and $\lambda_{SPP}$ were computed for different values of excitation wavelength $\lambda_{0}$ in the visible range (spanning from 400 to 700 nm) and for the following thickness values *t* of the metal: 36, 42, 48, 54, 60, 72, 84, 96 and 128 nm (see Figure 2). The produced data were used to train and then test a neural network architecture modelling two functions $\lambda_{SPP}\left(\lambda_{0},t\right)$ and $L_{SPP}\left(\lambda_{SPP},\lambda_{0},t\right)$, where $\lambda_{0}$ and *t* represent the excitation wavelength and the metal tickness, respectively. As previously stated, $\lambda_{SPP}$ and $L_{SPP}$ represent the propagation wavelength and length of the generated SPPs, respectively. Note that $L_{SPP}$ depends on $\lambda_{SPP}$, therefore the latter should be computed before the related $L_{SPP}$. However, the implemented NNNA is able to handle the intrinsic $L_{SPP}$ dependence on $\lambda_{SPP}$, therefore we have trained our NN to receive pairs $\left(\lambda_{0},t\right)$ as input, and return pairs $\left(\lambda_{SPP},L_{SPP}\right)$ as output.

After the training procedure, our NNNA has been tested by asking to produce the resulting $\lambda_{SPP}$ and $L_{SPP}$ when given inputs differing from the training set. The results of the NN have then been compared with the result given by the COMSOL simulations. The initial dataset computed by COMSOL consisted of 279 pairs $\left(\lambda_{0},t\right)$. Such a dataset has been shuffled randomly to avoid possible polarisation issues, then the resulting data points have been organised as a 1D input sequence.

In order to test our NNNA on the overall input space, we have organised the experiment in three phases, each dealing with its own set of input data. In each phase, firstly our NN starts from an untrained state and is given two thirds of the data derived by COMSOL as training set. The training phase lets our NN learn the respective input-output relations. Secondly, the trained NNNA is given the other one third of data, which was not used for training. Therefore, the NN gives results on data never seen before. Finally, for the last third of data an error is computed by comparing the NN results and the actual values given by COMSOL. The test goes on, for each phase repeating the said three steps, by having for each phase a different two third partition of data for training, and the remaining one third of data for test. Therefore, we have performed a blind cross validation on the overall input-output space, each time using data that were not fed to our NN during the training sequence on the same phase.

## 4. The Proposed Neural Network Architecture

The nature of the problem at hand presents several challenges that could tamper with both the performances and the precision of the results when trying to model such phenomena. The SPPs propagation length $L_{SPP}$ and wavelength $\lambda_{SPP}$ depend on each other, i.e., the propagation length intrinsically depends on the relative polariton’s wavelength *λ*. Therefore, the models for predictions of such values starting from *t* and $\lambda_{0}$ are non trivial, therefore we decided to cope with the intrinsic complexity of the model by using a Neural Network based approach to obtain a data-driven parametrical model [15,16,17,18,19,20].

**Figure 4:**
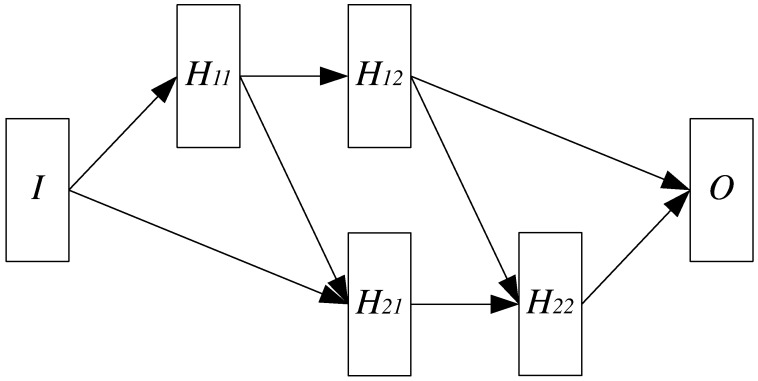
The proposed Nested Neural Network Architecture (NNNA).

In this work we propose a Nested Neural Network Architecture (NNNA) as a basis of our approach, due to its ability to cope with data interdependency problems such as the one at hand. The proposed NNNA is depicted in Figure 4 and consists of several stages, shown as rectangles. Overall, this architecture performs parallel asynchronous predictions of both $L_{SPP}$ and $\lambda_{SPP}$ by letting the stages inherent the prediction of $\lambda_{SPP}$, i.e., $H_{11}$ and $H_{12}$, to precede the respective stages inherent the prediction of $L_{SPP}$, i.e., $H_{21}$ and $H_{22}$, as can be seen by the interconnecting arrows.

This configuration achieves better performances when compared to a solution using two separate neural networks: the first to model and predict $\lambda_{SPP}$, and the second to obtain $L_{SPP}$ by taking as input the result obtained from the first neural network. For such a configuration, the two separate neural networks should be trained individually, then performance degradation would arise due to error composition among the two networks.

Previously, we had implemented an ad-hoc nested cascade network architecture and for it we had an accurate validation system that checks the performance status by considering the mean squared error, and the error introduced on the Fourier spectrum of the predicted signal [10,21]. The latter check was necessary in order to remove the possible artefacts introduced on the neural model used to predict $L_{SPP}$ by taking the results of the predictive neural model for $\lambda_{SPP}$. However, such a procedure is very expensive in terms of computational performance and overall elapsed time. Due to the interdependency of $\lambda_{SPP}$ and $L_{SPP}$, then, we have devised the proposed NNNA due to the possibility to train the neural system as an overall predictor for both $\lambda_{SPP}$ and $L_{SPP}$.

For the adopted NNNA, we can see that $\lambda_{SPP}$ and $L_{SPP}$ are being produced by two different pipelines, and while the first pipeline, producing $\lambda_{SPP}$, is autonomous, the second pipeline receives data from the first one. By using the data jointly for the two pipelines, giving them the same input, the second pipeline can output the correct value of $L_{SPP}$.

Figure 4 shows that the input layer *I* and the output layer *O* are distinct from other layers. Moreover, the hidden layers are organised as a shifted matrix, where the layers on the first row (namely $H_{11}$ and $H_{12}$) are involved on the prediction of $\lambda_{SPP}$, and the layers on the second row (namely $H_{21}$ and $H_{22}$) are involved on the prediction of $L_{SPP}$.

As stated before, in order to predict $L_{SPP}$, the correspondent layers need to have input from the layers predicting $\lambda_{SPP}$, therefore $H_{21}$ and $H_{22}$ are delayed by one time step with respect to $H_{11}$ and $H_{12}$. This is the reason behind the polygonal shape of the adopted neural network topology, which basically derives from a pair of Feed Forward Neural Networks (FFNNs) [22], with modified inputs and delayed time steps, each one dedicated to one prediction pipeline.

On the other hand, while the first pipeline $I\to H_{11}\to H_{12}\to O$ is a standard FFNN, the second pipeline $I\to H_{21}\to H_{22}\to O$ consists in a modified version of a FFNN where the input sources for $H_{21}$ and $H_{22}$ are twofold: the layer $H_{21}$ receives inputs both from the input layer *I* and the hidden layer $H_{11}$, the layer $H_{22}$ receives inputs from $H_{21}$ and $H_{12}$.

The internal composition detail of each layer for the proposed nested neural network architecture, as well as the activation functions and number of inputs is given in Table 1.

## 5. Training Procedure on OpenMP

The neural network architecture proposed above has introduced a sequential validation phase for the results of the first neural module. Validation has to be performed before the first module results can be sent as input for the second module. Unfortunately, such sequential operations make the training process expensive in terms of CPU time. In order to shorten training time we have developed a parallel implementation of the same neural network architecture, using OpenMP, that manages to obtain asynchronous training and validation.

Generally, when parallelising an application using OpenMP, processes are forked, joined and synchronised (e.g., by means of a barrier). Such mechanisms, however, introduce a runtime overhead, e.g., when the processes having produced and communicated their results have to wait until the synchronisation barrier is over. This is often the case when the computation times of processes are not perfectly balanced [23,24,25,26,27]. Therefore, our parallel version aims at reducing such an overhead by avoiding, as much as possible, the fork-join-barrier constructs, and by introducing instead processes that produce and consume data. The main reason for using OpenMP is that, by means of a shared memory, communication overhead among processes can be avoided, however, on the other hand, shared memory requires a complex handling of semaphores and locks before accessing some parts of the memory itself. We have handled the synchronisation concern in such a way that overhead is minimised [28].

Mainly, the proposed parallel solution is based on the continuous execution of different processes to care for the different phases of training for the above NNNA. In our experiments a multi-core processor has been used, however any kind of shared memory system supporting OpenMP directives can be employed. The first process is responsible to train the pipeline $I\to H_{11}\to H_{12}\to O$ and this process will be started at the beginning. The second process is responsible for training the pipeline $I\to H_{21}\to H_{22}\to O$, taking some input from the previous pipeline, and will be started after the first process has performed a given number of training epochs (see the following). The code of the second process has been developed so that it cannot change the weights of the connection between $H_{12}\to O$. The third process is responsible to train the stages $H_{12}\to O$ and $H_{22}\to O$, hence to make the output converge according to the given input *I* and the subsequent transformations by the other layers. Such a process is started after the second process has performed a given number of training epochs (see the following). Once the third process ends, the training continues with another iteration that starts with the first process, then the second, etc.

The proposed NNNA has been trained to predict the values of $\lambda_{SPP}$ and $L_{SPP}$ starting from an input vector.

(9) $$u\left(\tau \right)=\left[\lambda_{0},t\right]$$

To evaluate the performance of the NNNA two different kinds of errors were considered. We define two *local errors* $e_{1}$ and $e_{2}$, as well as a *global error* $e_{o}$ as follows:

(10) $$\begin{array}{cc} e_{1}= & \left|{\tilde{x}}^{\left(H_{12}\right)}-x^{\left(H_{12}\right)}\right|\\ e_{2}= & \left|{\tilde{x}}^{\left(H_{22}\right)}-x^{\left(H_{22}\right)}\right|\\ e_{o}= & \max\left\{e_{1},e_{2}\right\}\;\geq \;\left|{\tilde{x}}^{\left(O\right)}-x^{\left(O\right)}\right| \end{array}$$

where $\tilde{x}$ indicates the expected value (as given by COMSOL simulation) and x indicates the NNNA result. As can be clearly seen in Equation (10), the layer errors $e_{1}$ and $e_{2}$ have been computed as the module of the difference vector between the expected output vector and the output layer vector. Moreover, the global maximum error $e_{0}$ has been computed as the maximum error between the previous two errors. For each training epoch, the outputs from layers $H_{12}$, $H_{22}$ and *O* (see Figure 4) were used to compute the errors $e_{1}$, $e_{2}$ and $e_{o}$ as in Equation (10), respectively.

The training has been organised in four different phases executed on an OpenMP environment. The first phase provides as input to the whole NNNA a training pattern, which has been previously generated (i.e., the results of the COMSOL simulation). A fair amount of 50 epochs has been selected for this phase. The second phase starts once the first phase has terminated and uses a gradient descent algorithm to adjust the neural weights of the hidden layers $H_{11}$ and $H_{12}$ as if they would be an independent neural network. On the other hand, the latter training procedure takes into account only the predicted values of $\lambda_{SPP}$ excluding the evaluation of $L_{SPP}$. For this second phase 10 training epochs will be performed. Then, a third phase begins to adjust the weights of $H_{21}$ and $H_{22}$ basing on the error prediction of $L_{SPP}$. This third phase consists of 20 training epochs. Finally, in the fourth phase, the output layer weights will be adjusted.

The four phases above correspond each to a specific OpenMP process. At the end of the training epoch the global neural network performances are stored for further analysis. All the measures of performance involved in the training process are given by the Mean Squared Error (MSE), though for the global network performances, the formula is adjusted by using the global error $e_{o}$ in Equation (10).

Our effort during development has been to optimise the use of computational resources, hence autonomous processes needing as less synchronisation as possible have been implemented as described above. Our proposed solution manages to greatly reduce the wall-clock timeframe needed for the training.

## 6. Results and Conclusions

**Figure 5:**
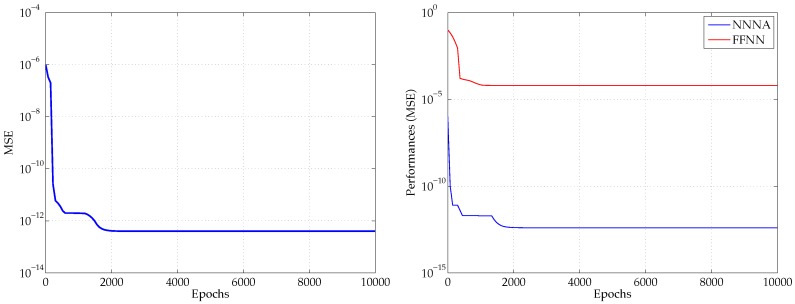
Global performance plot of the implemented Nested Neural Network Architecture (NNNA) (**left panel**), and a comparison with the performances obtained by using two separate Feed Forward Neural Networks (FFNNs) (**right panel**).

Figure 5 shows both a performance plot for the proposed NNNA, when trained with the adopted multithread technique on an OpenMP environment, and a comparison with a different approach using two standard FFNNs, each one having two hidden layers, respectively counting 10 and 7 neurons and activated by means of a *tansig* and a *logsig* function. The comparison shows that our proposed approach outperforms the FFNN approach, and the latter presents an error having a magnitude exceeding the predicted data values.

**Figure 6:**
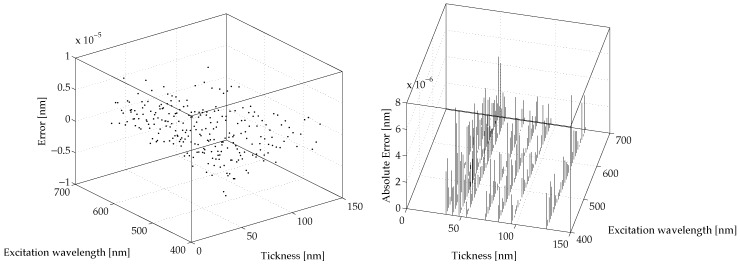
The obtained results: the model error $E_{\lambda_{SPP}}$ for the prediction of $\lambda_{SPP}$ computed as in Equation (11). The (**left**) shows the prediction error for $\lambda_{SPP}$ and the (**right**) shows its absolute value.

**Figure 7:**
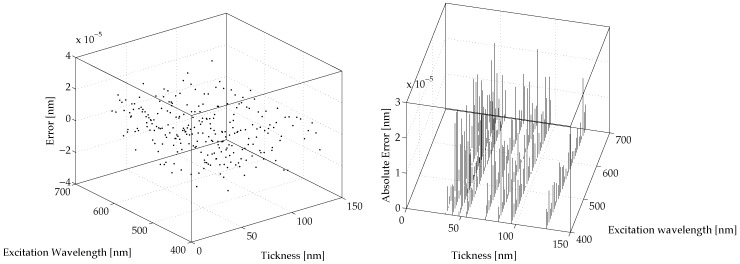
The obtained results: the model error $E_{L_{SPP}}$ for the prediction of $L_{SPP}$ computed as in Equation (11). The (**left**) shows the prediction error for $L_{SPP}$ and the (**right**) shows its absolute value.

Figure 6 and Figure 7 show the prediction errors for the proposed NNNA for the modeled values of $\lambda_{SPP}$ and $L_{SPP}$, respectively. The said prediction errors are computed as

(11) $$\begin{array}{cc} E_{\lambda_{SPP}}= & \left|{\tilde{\lambda }}_{SPP}-\lambda_{SPP}\right|\\ E_{L_{SPP}}= & \left|{\tilde{L}}_{SPP}-L_{SPP}\right| \end{array}$$

by difference between the results produced by COMSOL simulation and the results produced by our NNNA during testing (see Section 3).

For both $\lambda_{SPP}$ and $L_{SPP}$ the error is greatly lower than a realistic measurement a priori error. The average RMS for the predicted $\lambda_{SPP}$ is less than $7.55\times 10^{-9}$ nm, while the average RMS for the predicted $\lambda_{SPP}$ is less than $3.62\times 10^{-8}$ nm.

The proposed NNNA constitutes a novel approach to model the propagation of collective electrical oscillation at the interface between a metal and a dielectric. Our architecture has been able to model and correctly predict both the polaritons propagation wavelength $\lambda_{SPP}$ and the related propagation length $L_{SPP}$, as well as to reproduce the effective dependence of $L_{SPP}$ on $\lambda_{SPP}$. The latter outcome derives from both the adopted topology and the innovative training method. The NNNA consists of a comprehensive structure similar to a pair of cascade FFNNs, however it greatly improves the individual performances of singularly trained FNNN (see Figure 5).

This makes it possible to separately specialise each one of the two pipelines of the NNNA. While the first pipeline has been trained to predict $\lambda_{SPP}$, the second was dedicated to the prediction of $L_{SPP}$ also using the intermediate results produced by the first pipeline. Moreover, the adopted and dedicated training procedure is both advantageous in terms of computational performances, due to the multithread execution, and optimal for the training of the NNNA in order to model phenomena affected by a high degree of inner dependency.

Finally, the obtained errors, shown in Figure 6 and Figure 7, have positively validated the proposed techniques to model polaritons’ behaviour at a metal dielectric interface.

## Figures and Tables

**Table 1:** The internal composition of the proposed nested neural networks architecture.

Layer	*I*	$H_{11}$	$H_{12}$	$H_{21}$	$H_{22}$	*O*
Neurons	2	10	7	12	7	2
Inputs	-	2	10	12	17	14
Function	-	tansig	logsig	tansig	logsig	linear

## References

[1] Ozdemir S., Akhtar S., Gunal O.E., Khater M.E., Saritas R., Abdel-Rahman E.M., Yavuz M. (2015). Measuring the Quality Factor in MEMS devices. Micromachines.

[2] Franken R., Stolk R., Li H., Van der Werf C., Rath J., Schropp R. (2007). Understanding light trapping by light scattering textured back electrodes in thin film n-i-p-type silicon solar cells. J. Appl. Phys..

[3] Atwater H.A., Polman A. (2010). Plasmonics for improved photovoltaic devices. Nat. Mat..

[4] Fahr S., Rockstuhl C., Lederer F. (2009). Metallic nanoparticles as intermediate reflectors in tandem solar cells. Appl. Phys. Lett..

[5] Lutkenhaus J., Lowell D., George D., Zhang H., Lin Y. (2016). Holographic Fabrication of Designed Functional Defect Lines in Photonic Crystal Lattice Using a Spatial Light Modulator. Micromachines.

[6] Barrios C.A., Canalejas-Tejero V. (2016). Micro-Shaping of Nanopatterned Surfaces by Electron Beam Irradiation. Micromachines.

[7] Walters R.J., Van Loon R.V., Brunets I., Schmitz J., Polman A. (2010). A silicon-based electrical source of surface plasmon polaritons. Nat. Mat..

[8] De Waele R., Burgos S.P., Polman A., Atwater H.A. (2009). Plasmon dispersion in coaxial waveguides from single-cavity optical transmission measurements. Nano Lett..

[9] Barnes W.L., Dereux A., Ebbesen T.W. (2003). Surface plasmon subwavelength optics. Nature.

[10] Bonanno F., Capizzi G., Lo Sciuto G., Napoli C., Pappalardo G., Tramontana E. A Cascade Neural Network Architecture Investigating Surface Plasmon Polaritons Propagation for Thin Metals in OpenMP. Proceedings of International Conference on Artificial Intelligence and Soft Computing (ICAISC).

[11] Zia R., Selker M.D., Catrysse P.B., Brongersma M.L. (2004). Geometries and materials for subwavelength surface plasmon modes. JOSA A.

[12] Shah A., Torres P., Tscharner R., Wyrsch N., Keppner H. (1999). Photovoltaic technology: The case for thin-film solar cells. Science.

[13] Dawson P., De Fornel F., Goudonnet J. (1994). Imaging of surface plasmon propagation and edge interaction using a photon scanning tunneling microscope. Phys. Rev. Lett..

[14] Chudnovsky E.M. (2007). Theory of spin Hall effect: Extension of the Drude model. Phys. Rev. Lett..

[15] Haykin S. (2004). Neural Networks: A comprehensive foundation.

[16] Bonanno F., Capizzi G., Napoli C. Hybrid neural networks architectures for SOC and voltage prediction of new generation batteries storage. Proceedings of IEEE international conference on clean electrical power (ICCEP).

[17] Napoli C., Pappalardo G., Tina G.M., Tramontana E. (2015). Cooperative Strategy for Optimal Management of Smart Grids by Wavelet RNNs and Cloud Computing. IEEE Transactions on Neural Networks and Learning Systems.

[18] Paliwal M., Kumar U.A. (2009). Neural networks and statistical techniques: A review of applications. Expert Syst. Appl..

[19] Capizzi G., Bonanno F., Napoli C. Recurrent neural network-based control strategy for battery energy storage in generation systems with intermittent renewable energy sources. Proceedings of IEEE International Conference on Clean Electrical Power (ICCEP).

[20] Gotleyb D., Lo Sciuto G., Napoli C., Shikler R., Tramontana E., Woźniak M. Characterisation and Modeling of Organic Solar Cells by Using Radial Basis Neural Networks. Proceedings of International Conference on Artificial Intelligence and Soft Computing (ICAISC).

[21] Bonanno F., Capizzi G., Coco S., Napoli C., Laudani A., Lo Sciuto G. Optimal thicknesses determination in a multilayer structure to improve the SPP efficiency for photovoltaic devices by an hybrid FEM—Cascade Neural Network based approach. Proceedings of IEEE International Symposium on Power Electronics, Electrical Drives, Automation and Motion (SPEEDAM).

[22] Mandic D.P., Chambers J. (2001). Recurrent Neural Networks for Prediction: Learning Algorithms, Architectures and Stability.

[23] Dagum L., Enon R. (1998). OpenMP: An industry standard API for shared-memory programming. IEEE Comput. Sci. Eng..

[24] Bonanno F., Capizzi G., Lo Sciuto G., Napoli C., Pappalardo G., Tramontana E. A novel cloud-distributed toolbox for optimal energy dispatch management from renewables in igss by using wrnn predictors and gpu parallel solutions. Proceedings of IEEE International Symposium on Power Electronics, Electrical Drives, Automation and Motion (SPEEDAM).

[25] Calvagna A., Tramontana E. Delivering dependable reusable components by expressing and enforcing design decisions. Proceedings of IEEE Computer Software and Applications Conference Workshops (COMPSACW).

[26] Giunta R., Pappalardo G., Tramontana E. Superimposing roles for design patterns into application classes by means of aspects. Proceedings of the ACM Symposium on Applied Computing (SAC).

[27] Bannò F., Marletta D., Pappalardo G., Tramontana E. Tackling consistency issues for runtime updating distributed systems. Proceedings of IEEE International Symposium on Parallel and Distributed Processing, Workshops and Phd Forum (IPDPSW).

[28] Chapman B., Jost G., Van Der Pas R. (2008). Using OpenMP: Portable Shared Memory Parallel Programming.

